# Nerolidol/hydroxypropyl-*beta*-cyclodextrin inclusion complex nanofibers: Active food packaging for effective strawberry preservation

**DOI:** 10.1016/j.fochx.2025.102584

**Published:** 2025-05-26

**Authors:** Yu Zhang, Fengrui Li, Honglei Yan, Yan Zhang, Shimiao Feng, Lei Deng, Lixia Zhao, Shuang Gao, Ying Fu, Fei Ye

**Affiliations:** Department of Chemistry, Northeast Agricultural University, Harbin 150030, China

**Keywords:** Electrospinning, Antioxidant activity, Antimicrobial activity, Fruit preservation, Shelf life extension

## Abstract

The development of bio-based active food packaging is urgently needed to address food safety concerns. In this study, nerolidol/hydroxypropyl-*β*-cyclodextrin inclusion complex nanofibers (Nerolidol/HP*β*CD-IC-NF) were prepared by electrospinning to improve the thermal stability and water solubility of natural antimicrobial nerolidol. Structural characterization (FTIR, XRD, ^1^H NMR) confirmed successful nerolidol encapsulation within HP*β*CD cavities. The resulting bead-free nanofibers exhibited high surface area and porosity. Nerolidol/HP*β*CD-IC-NF demonstrated a 3.3-fold increase in free radical scavenging capacity compared to free nerolidol, along with potent antimicrobial activity (88.1 % and 76.0 % inhibition against *E. coli* and *S. aureus*, respectively). In strawberry preservation trials, nanofiber membranes reduced *B. cinerea* lesion diameters by 5.56-fold after 5 days compared to untreated controls, significantly extending shelf life. These findings highlight Nerolidol/HP*β*CD-IC-NF as a promising solution for preventing microbial contamination and improving food preservation efficacy in active packaging applications.

## Highlights

• Nerolidol was successfully encapsulated in HP*β*CD electrospinning nanofibers.
• Nerolidol/HP*β*CD-IC-NF enhanced the thermal stability of nerolidol.
• Nerolidol/HP*β*CD-IC-NF exhibited excellent antioxidant and antimicrobial properties.
• Nerolidol/HP*β*CD-IC-NF excelled in prolonging strawberry preservation time.

## 1 Introduction

With the increasing awareness of consumers, food safety is getting more and more attention (Zhang, Jiang, et al., 2022). It is worth exploring to develop food preservation technology (Deshmukh & Gaikwad, 2024). Compared with artificial chemical additives, active food packaging containing natural antibacterial agents and antioxidants has extended the shelf life and safety of food (Atta et al., 2022). The effective utilization of active compounds is particularly important in the design of active food packaging. Nerolidol is a natural sesquiterpene alcohol, existing in various plant essential oils, and has a unique aroma (Chan et al., 2016; Meeran et al., 2021). Its main biological activities include antioxidant, antimicrobial, anticancer, and anti-inflammatory properties, which are relevant to active food packaging (Balakrishnan et al., 2022; de Moura et al., 2021; Trindade et al., 2020). It has been confirmed that it has sufficient safety in food additives by the U.S. Food and Drug Administration (FDA) (Schepetkin et al., 2022). Baldisserotto et al. investigated the antioxidant properties of nerolidol as a food additive in a biological model system. The antioxidant capacity of fish with nerolidol was effectively improved against peroxyl free radicals, suggesting its potential to extend the shelf life of food products (Baldissera et al., 2020). However, the low water solubility and instability of nerolidol limit its effective utilization. Therefore, it is crucial to seek an efficient encapsulation material for the application of nerolidol in active food packaging.

There are many efficient encapsulation materials in the field of active food packaging. Polymer-based materials, as the mainstream encapsulation materials, are putting great pressure on the global environment due to the difficulty of degradation (Stoica et al., 2024). Therefore, bio-based polymer-free active food packaging materials are gaining popularity as an alternative to traditional polymer-based materials (Chausali et al., 2022). 2-Hydroxypropyl-*β*-cyclodextrin (HP*β*CD) was etherified to significantly improve the water solubility and the encapsulation efficiency of natural cyclodextrins (Gao et al., 2021). HP*β*CD featured a hydrophilic exterior and a hydrophobic cavity, facilitating their interaction with a broad range of guest molecules (Rajamohan et al., 2023). In the design process of food packaging, the toxicity of encapsulated material is particularly crucial. HP*β*CDs are approved by the FDA for use as food additives, which indicates that the toxicity of HP*β*CD is recognized within the recommended use levels (Becktel et al., 2022; El Darzi et al., 2021; Mehta et al., 2022). Hu et al. successfully prepared HP*β*CD/epigallocatechin inclusion complexes using ultrasonic method and prepared nanofibers for fruit preservation (Hu, Huang, et al., 2024). Nonetheless, how to quickly and efficiently prepare functional nanofibers remains a challenge.

Nanofibers fabricated *via* electrospinning technology exhibit advantages such as fine fiber diameter, large specific surface area, and high porosity, which has found extensive applications in diverse fields (Narayanan et al., 2018; Topuz & Uyar, 2020). These characteristics can enhance the effective utilization of active substances for the purpose of facilitating broader applications of nanofibers in the delivery of active substances (Aytac et al., 2017). According to previous reports, the physicochemical properties of active substances are significantly enhanced when nanofibers are prepared after the formation of inclusion complexes with cyclodextrins. Notably, Gao et al. used nanofibers loaded with betulin, which significantly improved the water solubility and thermal stability of botulin (Zhang et al., 2024). Uyar et al. prepared carvacrylaldehyde/cyclodextrin inclusion-loaded gelatin/purulan nanofibers by electrostatic spinning method, which enhanced their properties such as antimicrobial and antioxidant properties for use in active food packaging (Ertan et al., 2023). It is therefore reasonable to hypothesize that the antimicrobial and antioxidant properties of nerolidol can be enhanced by fabricating nanofibers through electrospinning technology to extend the shelf life of food products.

In this work, the inclusion complex nanofibers with an equal molar ratio of nerolidol to HP*β*CD were successfully fabricated *via* electrospinning for active food packaging. The microscopic morphology of the nanofibers was observed in detail using scanning electron microscopy (SEM). The structure was characterized by nuclear magnetic resonance hydrogen spectroscopy (^1^H NMR), Fourier transform infrared spectroscopy (FTIR) and X-ray diffraction (XRD) methods. Meanwhile, its thermal stability and solubility were evaluated by thermogravimetric analysis (TGA) and phase solubility studies. In addition, the antioxidant activity of Nerolidol/HP*β*CD-IC-NF was further evaluated, as well as *in vitro* and *in vivo* antimicrobial activity. This work significantly improved the stability and bioavailability of nerolidol and proposed an effective strategy for active food packaging by preparing nanofiber by electrospinning technique.

## 2 Materials and methods

### 2.1 Materials

Nerolidol (≥96 %, molecular weight: 222.37) was supplied by Yuanye Biotechnology Co., Ltd. (Shanghai, China). HP*β*CD (≥98 %, molecular formula: C_51_H_88_O_38_, molecular weight ∼ 1541) was sourced from Zhiyuan Biochemical Co., Ltd. (Shandong, China). Potassium bromide (≥99 %, molecular weight: 119.00) was purchased from Guangfu Co., Ltd. (Tianjin, China). Ethanol was obtained from Tianli Chemical Reagent Co., Ltd. (Tianjin, China). Dimethyl sulfoxide-*d*_6_ (DMSO‑*d*_6_) was sourced from Tianli Chemical Reagent Co., Ltd. (Tianjin, China). *Escherichia coli* (*E. coli*), *Staphylococcus aureus* (*S. aureus*)*,* and Botrytis cinerea (*B. cinerea*) were obtained from Biological Resource Collection Center (Shanghai, China). Strawberries purchased from Beauty Supermarket (Harbin, China). Agar medium was supplied by Aoboxing Biotechnology Co., Ltd. (Beijing, China). DPPH and all other chemical reagents used in this study were obtained from Aladdin (Shanghai, China).

### 2.2 Preparation of inclusion complex solutions

2 g of HP*β*CD was added to 1 mL of distilled water and stirred continuously at room temperature until it dissolved to obtain an aqueous solution of HP*β*CD with 200 % concentration. An oily liquid of nerolidol equal to the number of moles of HP*β*CD was mixed into the above HP*β*CD solution and stirred at room temperature for 24 h to obtain a solution of the inclusion complex. Additionally, the HP*β*CD solution without nerolidol was prepared with the same concentration as a control.

### 2.3 Electrospinning of Nerolidol/HP*β*CD-IC-NF and HP*β*CD-NF

The electrostatic spinning process was slightly adapted from the method of Amjadi et al. (Ahangari et al., 2025). Fix the tin foil on the collector. Inject the spinning solution into a 5 mL syringe with a 21-gauge needle. Push the pump at a flow rate of 0.5 mL/h. Electrospinning distance and voltage were set to 15 cm and 15 kV, respectively. The electrospinning process was carried out in a closed environment at ambient temperature (25 °C) and humidity (18 %).

### 2.4 Determination of viscosity and conductivity of solutions

The viscosity and dielectric constants of HP*β*CD and Nerolidol/HP*β*CD inclusion complex solutions at ambient temperature were measured using the NDJ-8S rotational viscometer and the DDSJ-308F conductivity meter, respectively (Gao et al., 2023).

### 2.5 Morphological analysis

HP*β*CD nanofibers (HP*β*CD-NF) and Nerolidol/HP*β*CD-IC-NF appearance and morphology were observed using field emission SEM (SU-8010, Hitachi, Japan) with a voltage acceleration of 12.5 kV. The nanofiber sample was affixed to the SEM holder and subsequently coated with a thin layer of gold. Then 100 nanofibers were randomly selected based on different positions in the SEM images, fiber diameter values were measured using Nano Measurer software and the average fiber diameter was calculated (Feng et al., 2024).

### 2.6 ^1^H NMR analysis

^1^H NMR spectra of Nerolidol, HP*β*CD-NF, and Nerolidol/HP*β*CD-IC-NF were recorded at AVANCE NEO 400 MHz spectrometer (BRUKER, Beijing, China). DMSO‑*d*_6_ and TMS are used as solvent and internal standard, respectively. The ^1^H NMH spectra results were analyzed by MestReNova (Song et al., 2021).

### 2.7 FT-IR analysis

Nerolidol, HP*β*CD-NF, and Nerolidol/HP*β*CD-IC-NF were analyzed using an Alpha-T infrared spectrometer (Bruker VERTEX 70). The samples were mixed with KBr powder at 1:100 and pressed into transparent flakes. To record the chemical structure of the analyzed samples, the spectra were obtained with a resolution of 4 cm^−1^, spanning from 4000 to 400 cm^−1^, with 16 scans per sample (Amjadi et al., 2022).

### 2.8 XRD analysis

HP*β*CD-NF and Nerolidol/HP*β*CD-IC-NF were analyzed using a Philips X-ray diffractometer (Malvern Panalytic, The Netherlands). The instrument voltage was set to 40 kV and the current to 30 mA. The X-ray diffractometer was adjusted to emit scanning at a rate of 2°/min using C*u*-K*α* radiation in the range of 5–70° (Amjadi et al., 2024).

### 2.9 TGA analysis

The thermal stability of nerolidol, HP*β*CD-NF, and Nerolidol/HP*β*CD-IC-NF were performed by the thermogravimetric analyzer. The sample (approximately 5 mg) were loaded into a crucible with a 4 mm inner diameter. The TGA tests were conducted within a temperature range of 20–600 °C and a heating rate of 10 °C/min, while maintaining a constant nitrogen flow rate of 50 mL/min (Topuz et al., 2020).

### 2.10 Phase solubility studies

Ten 25 mL portions of an aqueous solution of HP*β*CD at concentrations of 0–10.00 mmol/L were prepared, ensuring that each concentration was mixed well. 0.625 mL of nerolidol was added to each HP*β*CD aqueous solution. The conical flasks containing the mixed solutions were shaken in a constant temperature water bath shaker for 72 h (150 rpm, 37 °C). The mixed solution was filtered through a 0.45 μm microporous filter membrane to remove undissolved nerolidol particles and other possible impurities. The concentration of nerolidol in solution was measured using a UV spectrophotometer. UV spectroscopy measured absorbance at 210 nm using a quartz cuvette with anhydrous ethanol as a blank. The experiment was repeated three times to ensure the reliability of the data. The solubility of nerolidol at each concentration was calculated from the measured UV absorbance and the phase solubility graph was plotted. The horizontal axis is the concentration of HP*β*CD (mmol/L) and the vertical axis is the solubility of nerolidol. The equilibrium constant (*K*s) was computed using the following eq. (1):

(1) $$K_{s}=\frac{k}{b\left(1-k\right)}$$

where *k* indicates the slope of the phase solubility curve and *b* indicates the solubility of nerolidol.

Complexation efficiency (*CE*) was calculated using the following eq. (2):

(2) $$\mathrm{CE}=b\times K_{s}=\frac{\left[\mathrm{Nerolidol}/\mathrm{HP}\beta \mathrm{CD}\right]}{\left[\mathrm{HP}\beta \mathrm{CD}\right]}=\frac{k}{1-k}$$

where [*Nerolidol*/HP*β*CD] indicates the concentration of nerolidol and [HP*β*CD] indicates the concentration of HP*β*CD.

### 2.11 Rapid dissolution test

A 20 × 20 mm piece of Nerolidol/HP*β*CD-IC-NF was cut and placed in a Petri dish for rapid dissolution test. 1 mL of deionized water was added drop by drop to the petri dish, followed by photographs to record the water solubility of the samples (Yang et al., 2024).

### 2.12 Molecular simulation

Molecular simulations were performed using SYBYL-X 2.0 software (Tripos, St. Louis, MO, USA). The software was used to optimize and create 3D structures of nerolidol and HP*β*CD molecules. The Gasteiger-Huckel charges of the two molecules were calculated simultaneously (Leng et al., 2023). Accurate modeling using Discovery Studio 2.5 (Accelrys Software, San Diego, CA, USA) was used to predict the encapsulation patterns of HP*β*CD and Nerolidol. The CHARMm force field was applied to the HP*β*CD molecule. We defined the active site within cavity of HP*β*CD as a sphere, specifying a radius of 13 Å, to identify the ligand's atoms accurately (Li et al., 2025). The rest of the parameters are left at their default values.

### 2.13 Mechanical properties

The elongation at break (EAB) and tensile strength (TS) of the nanofiber membrane (50 mm × 10 mm) were measured using a universal testing machine. The tests were performed at a tensile speed of 1 mm/min. Each sample was repeated three times and the results were expressed as mean ± standard deviation.

### 2.14 Cytotoxicity assay

The biocompatibility of nanofibers was determined by CCK-8 kit. NIH 3 T3 cells were inoculated into sterile 96-well plates and incubated at 37 °C for 24 h (5 % CO_2_). The culture medium of each well was discarded from the sterile 96-well plate after 24 h of cell adherence. The cells were then treated with different concentrations of nanofibers (10, 100, 500, 1000, 2000 and 5000.00 μg/mL) for 24 h. Finally, 10 μL of CCK-8 color solution was added to each well and the incubation was continued for 4 h. The cells were then incubated at 37 °C for 24 h. The cells were then incubated at 37 °C for 24 h. Absorbance at 450 nm was measured by a microplate reader. Cell viability was calculated according to eq. (3):

(3) $$\mathrm{Cell viability}=\frac{\mathrm{OD}-{\mathrm{OD}}_{0}}{{\mathrm{OD}}_{1}-{\mathrm{OD}}_{0}}\times 100\%$$

where, OD is the absorbance of the experimental sample group, OD_0_ is the absorbance of the blank group and OD_1_ is the absorbance of the no sample control group.

### 2.15 Antioxidant activity analysis

The antioxidant activities of nerolidol, HP*β*CD-NF, and Nerolidol/HP*β*CD-IC-NF were evaluated based on previous report (Gao et al., 2022). Through the DPPH free radical scavenging experiment, the solution with different samples changed from dark purple to yellow, which represented the neutralization of DPPH free radicals, and the degree of discoloration depends on the antioxidant activity. A total of 5 mL was prepared by dissolving 10^−4^ mol/L DPPH solution in water/ethanol (1:1) as a solvent. In addition, 10 *μ*L nerolidol, Nerolidol/HP*β*CD-IC-NF (with the same content of nerolidol in nerolidol sample), and HP*β*CD-NF with the equivalent weight of Nerolidol/HP*β*CD-IC-NF were dissolved in the prepared DPPH solution, respectively. The mixed solution was kept at room temperature in dark for 1 h. The UV absorbance of the samples at 517 nm was recorded. The resulting samples were calculated using eq. (4):

(4) $$\mathrm{Scavenging of DPPH}\;\left(\%\right)=\frac{A_{c}-A_{s}}{A_{c}}\times 100\%$$

where *A*c indicates the absorbance of the DPPH solution and *A*s indicates the absorbance of the sample after the reaction.

### 2.16 *In vitro* antibacterial assay

The *in vitro* antibacterial activity of HP*β*CD-NF and Nerolidol/HP*β*CD-IC-NF against *E. coli* and *S. aureus* was evaluated by colony counting method by previously reported literature (Gao et al., 2022). Bacteria in Luria-Bertani medium were incubated at 37 °C for 12 h to obtain a concentration of approximately 1 × 10^8^ CFU/mL after which they were diluted to 1 × 10^7^ CFU/mL.50 mg of nanofibers were taken in bacterial PBS solution and after incubation, the bacterial solution was spread on agar plates. The colonies were counted by microscope and three sets of parallel experiments were performed. Based on the counting results (mean ± standard deviation), the antibacterial efficiency of the samples was computed by the eq. (5):

(5) $$\mathrm{Bacteriostatic efficiency}\;\left(\%\right)=\frac{C-T}{C}\times 100\%$$

where *C* and *T* represent the number of bacteria in the HP*β*CD-NF and Nerolidol/HP*β*CD-IC-NF groups, respectively.

### 2.17 *In vivo* antifungal assay

This study investigated the *in vivo* antifungal properties of nanofiber membranes on strawberries. Strawberries of uniform size and maturity were selected to create a wound of 1 mm in diameter on the same part of the strawberry. Next, 10 μL of *B. cinerea* solution at a concentration of 1 × 10^6^ CFU/mL was taken and inoculated onto the wound. 10 mg of HP*β*CD-NF, 5 μL of nerolidol, and Nerolidol/HP*β*CD-IC-NF containing 5 *μ*L of nerolidol were weighed and attached to the wound. The treated strawberries were incubated in a mold incubator for 5 days (27 °C, 90 % relative humidity). During this period, the lesions were photographed and recorded every 24 h (Gao, Zhang, et al., 2025). The diameter of the lesions was calculated using Nano Measurer 1.2 software. Each set of samples was repeated three times.

### 2.18 Application of nanofiber membrane in strawberry preservation

Determination of weight loss of strawberries during storage. Strawberries were weighed daily and each group was repeated three times. The rate of weight loss of strawberries was determined according to the following eq. (6):

(6) $$\mathrm{Weight loss}\;\left(\%\right)=\frac{W_{1}-W_{2}}{W_{1}}\times 100\%$$

where *W*_*1*_ is the initial weight of the strawberry; *W*_*2*_ is the weight of the strawberry on the sampling day.

The pH difference method was used to determine the total anthocyanin content of strawberries. Slightly modified from the method of Lee et al. (Lee et al., 2005). The strawberry samples were thoroughly mixed with acid ethanol, and then the extracts were centrifuged at room temperature for 20 min at 4000 r/min. The supernatants were taken and diluted with KCI buffer at pH 1.0 and CH_3_COONa buffer at pH 4.5, respectively. The absorbance of the dilutions was measured at 520 nm and 700 nm. Anthocyanin concentrations were calculated based on the molecular weights of geranylgeranyl-3-glucoside (P-3-G) molar extinction coefficients of 22,400 and 433 g/mol. Measurements were repeated three times and the average value reported. The following eq. (7) was used to calculate the anthocyanin content:

(7) $$\mathrm{TA}=\frac{A\times 433\times V}{22400\times m}\times 100$$

where *TA* is the total anthocyanin concentration; *A = (A*_*520 nm*_ *- A*_*700 nm*_*)* pH 1 *- (A*_*520 nm*_ *- A*_*700 nm*_*)* pH 4.5; *V* is the volume of the extract; *m* is the strawberry fresh weight.

### 2.19 Statistical analysis

Statistical analysis was performed using IBM SPSS Statistics 27 software (Chicago, USA). All data were analyzed by one-way analysis of variance (ANOVA). The results of each experiment were expressed as mean ± standard deviation and repeated three times.

## 3 Results and discussion

### 3.1 Morphology analysis

**Fig. 1:**
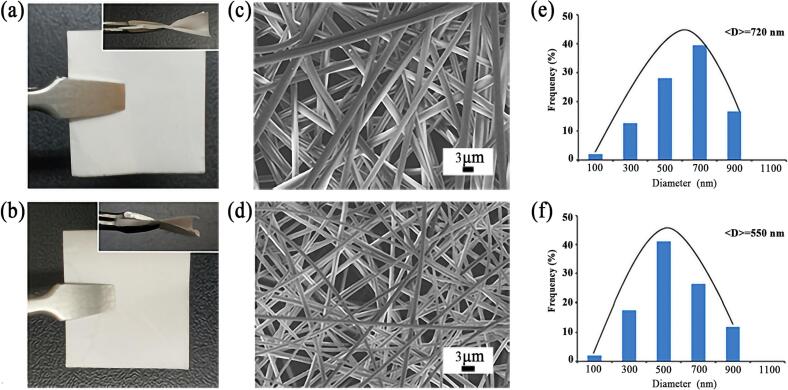
The physical photos of HP*β*CD-NF (a) and Nerolidol/HP*β*CD-IC-NF (b); SEM images of HP*β*CD-NF (c) and Nerolidol/HP*β*CD-IC-NF (d); and AFD distributions of HP*β*CD-NF (e) and Nerolidol/HP*β*CD-IC-NF (f).

The morphology photos, SEM images, and average fiber diameter (AFD) distribution plots of HP*β*CD-NF and Nerolidol/HP*β*CD-IC-NF are illustrated in Fig. 1. The morphology photos depict the visual characteristics of the nanofiber membranes of HP*β*CD-NF and Nerolidol/HP*β*CD-IC-NF. The nanofiber membranes of both types exhibited a uniform and dense fiber structure, characterized by smooth surfaces devoid of any discernible defects. The prepared nanofibers were nano-sized, high surface area, and beadless, which were shown by SEM images. The electrospinning process was conducted using optimized parameters, resulting in nanofiber membranes exhibiting favorable fiber spacing, morphology. The viscosity and conductivity of the electrospinning solution are crucial factors affecting fiber formation. The viscosity and conductivity of the HP*β*CD solution were measured be 309.3 ± 28.1 mPa·S and 28.50 ± 0.25 μS/cm, respectively. The viscosity of the Nerolidol/HP*β*CD-IC solution, in contrast, exhibited a higher value of 201.9 ± 16.8 mPa·S, while its conductivity was comparatively lower be 32.6 ± 0.91 μS/cm. Typically, viscosity determines the flow and stretchability of the solution during electrospinning, while conductivity exerts influence on charge transfer and fiber formation throughout the spinning process (Uyar & Besenbacher, 2008).The calculations showed that the AFD of HP*β*CD-NF and Nerolidol/HP*β*CD-IC-NF were 720 ± 125 nm and 550 ± 102 nm, respectively. The changes in AFD of the two fibers were associated with changes in solution viscosity and conductivity. The Nerolidol/HP*β*CD-IC solution had lower viscosity and higher conductivity compared to the HP*β*CD solution. Gao et al. proposed that the lower viscosity and higher conductivity of the spinning solution led to a decrease in fiber diameter (Gao et al., 2025). This might be the reason why the AFD of Nerolidol/HP*β*CD-IC-NF was slightly lower than that of HP*β*CD-NF.

### 3.2 ^1^H NMR analysis

**Fig. 2:**
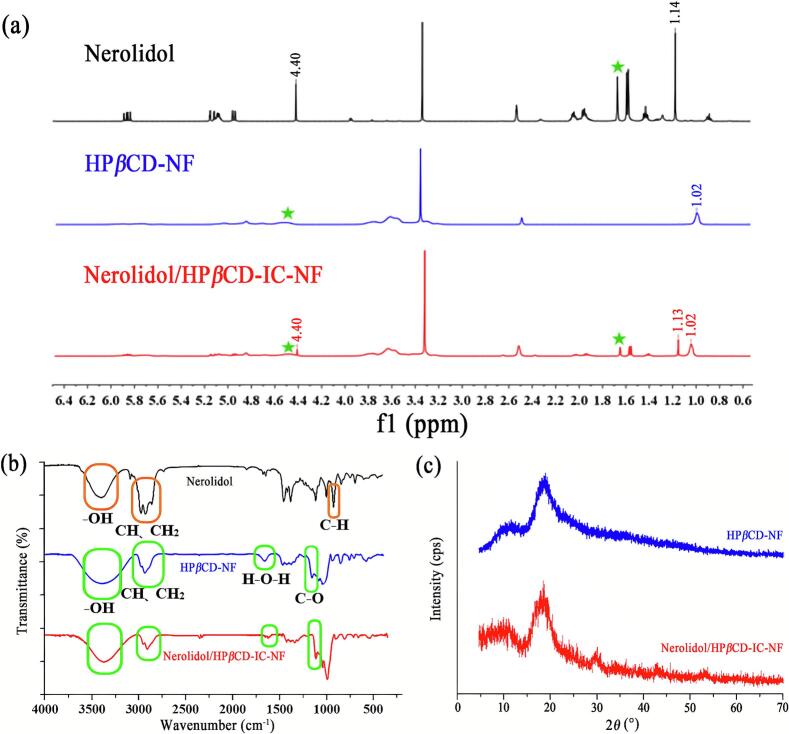
^1^H NMR spectra of nerolidol, HP*β*CD-NF, and Nerolidol/HP*β*CD-IC-NF (a); FTIR spectra of nerolidol, HP*β*CD-NF, and Nerolidol/HP*β*CD-IC-NF (b); XRD spectra of HP*β*CD-NF and Nerolidol/HP*β*CD-IC-NF (c).

^1^H NMR spectroscopy is a pivotal technique for substantiating the formation of inclusion complexes, while also enabling the determination of the stoichiometric ratio between guest nerolidol and host HP*β*CD through peak area analysis (Celebioglu & Uyar, 2020). The ^1^H NMR spectroscopy of nerolidol, HP*β*CD-NF, and Nerolidol/HP*β*CD-IC-NF are illustrated in Fig. 2 (a). The 4.40 ppm aldehyde hydrogen peak in nerolidol was significantly reduced in the Nerolidol/HP*β*CD-IC-NF spectrum, indicating encapsulation of the aldehyde group. However, the characteristic peak of the methyl hydrogen connected to the aldehyde carbon at 1.14 ppm in nerolidol remains detectable, indicating that part of methyl structure of nerolidol was exposed outside the HP*β*CD cavity. Additionally, the characteristic peak of HP*β*CD at 1.02 ppm was observed in both HP*β*CD-NF and Nerolidol/HP*β*CD-IC-NF. Based on these results, Nerolidol/HP*β*CD-IC had been successfully formed which was effectively demonstrated. By selecting non-overlapping positions of nerolidol and HP*β*CD-NF in the ^1^H NMR spectra, integration calculations were performed to determine the relative intensities of characteristic hydrogen peaks. The stoichiometric ratio between HP*β*CD (1.03 ppm) and nerolidol (1.63 ppm) was calculated to be 1:0.90, indicating a high encapsulation efficiency of 90 % for nerolidol within HP*β*CD. Gao et al. prepared nanofibers of betulin and HP*β*CD inclusion complex with a stoichiometric ratio of 1:2 by electrospinning technique (Gao, Zhang, et al., 2025). The present study of the inclusion complex obtained nearly 1:1 stoichiometric ratio, which proved that the inclusion complex system had higher encapsulation efficiency. And this result was similar to the findings of Uyar et al. who prepared ciprofloxacin/cyclodextrin inclusion complex nanofibers with a stoichiometric ratio of 1:1 (Aytac et al., 2019). Therefore, the above results further validated that HP*β*CD could encapsulate nerolidol efficiently and was capable of loading a higher amount of active substances.

### 3.3 FTIR analysis

FTIR spectra are widely utilized to evaluate the interactions between host and guest molecules in inclusion complexes. The analysis of the shape and frequency changes of characteristic absorption peaks corresponding to functional groups provides valuable insights into the encapsulation status of host-guest molecules. The FTIR spectra of nerolidol, HP*β*CD-NF, and Nerolidol/HP*β*CD-IC-NF are illustrated in Fig. 2 (b). In the FTIR spectrum of nerolidol, the absorption peak at 3392 cm^−1^ corresponds to -OH. The strong absorption peaks at 2968 cm^−1^ and 2925 cm^−1^ represent -CH and -CH_2_, respectively. The stretching vibration peak of C—H in the olefin was observed at 920 cm^−1^. HP*β*CD-NF had a broad vibration of the -OH functional group (3410 cm^−1^) and characteristic absorption peak of -CH and -CH_2_ vibrations (2925 cm^−1^). The vibration peak at 1646 cm^−1^ and 1015 cm^−1^ represented H-O-H and C—O, respectively. Due to the shielding effect of HP*β*CD, the characteristic peaks of nerolidol in the FTIR spectra of Nerolidol/HP*β*CD-IC-NF were attenuated to different degrees. Combined with the ^1^H NMR spectrum, the FTIR spectrum of the samples helped to confirm the presence of nerolidol in Nerolidol/HP*β*CD-IC-NF, further validating the formation of the Nerolidol/HP*β*CD inclusion complex.

### 3.4 XRD analysis

XRD serves as an effective tool for analyzing crystallographic information in nanofibers. The XRD data of nerolidol could not be obtained because of its liquid state at room temperature. The XRD spectra of HP*β*CD-NF and Nerolidol/HP*β*CD-IC-NF are presented in Fig. 2 (c). As depicted, HP*β*CD-NF displayed broad and diffuse diffraction peaks, indicating an amorphous structure. In addition, the XRD pattern of Nerolidol/HP*β*CD-IC-NF exhibited a significant weakening of the diffraction peaks. This proved the compound presented an amorphous state, suggesting that nerolidol may have influenced the crystal structure of HP*β*CD in its encapsulation. In summary, there was an increase in amorphous structures, which confirmed the formation of inclusion complex containing nerolidol and HP*β*CD. Furthermore, the results of Narayanan et al. showed that one of the effective means of improving the poor aqueous solubility of a drug is for the drug to be non-crystallization (Narayanan et al., 2017). In this study, nerolidol was unable to form crystalline aggregates because it was separated by its cavity due to hydrogen bonding and hydrophobicity of HP*β*CD. Eventually, nerolidol is in an amorphous state, which improves its dispersion and water solubility in the system. There was an effective interaction between nerolidol and HP*β*CD significantly changed its structural properties, which was consistent with the results of ^1^H NMR and FTIR spectroscopy.

### 3.5 TGA analysis

**Fig. 3:**
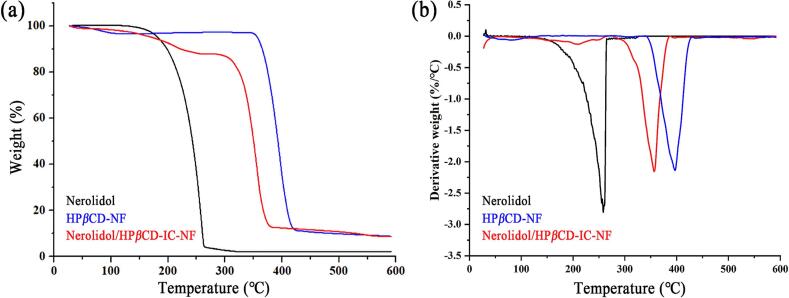
TGA curves (a) and DTG curves (b) of nerolidol, HP*β*CD-NF and Nerolidol/HP*β*CD-IC-NF.

Thermogravimetric analysis (TGA) is employed to assess the thermal stability of a sample by monitoring variations in its mass. The TGA curves of nerolidol, HP*β*CD-NF, and Nerolidol/HP*β*CD-IC-NF are illustrated in Fig. 3 (a). The improvement of the thermal stability of nerolidol can effectively prevent its volatilization to enhance its bioavailability. The TGA curve of nerolidol exhibited a single mass loss process occurring between 156 and 252 °C due to the volatilization of the essential oil. The mass loss process of HP*β*CD exhibited two distinct stages. The first stage involved the release of crystalline water primarily occurring below 100 °C, while the second stage occurring between 344 and 425 °C, ascribed to the thermal decomposition of HP*β*CD itself. Nerolidol/HP*β*CD-IC-NF displayed a unique mass loss profile compared to the other samples, which was assigned to the loss of crystalline water below 100 °C, the volatilization of nerolidol essential oil at 195–285 °C, and the decomposition of HP*β*CD at 344–398 °C, respectively. The volatilization temperature of nerolidol in Nerolidol/HP*β*CD-IC-NF was higher than that of pure nerolidol. The DTG results obtained from the first derivative of the TGA data are presented in Fig. 3 (b). The peak positions of the DTG corresponded with the weight loss observed in the TGA. In the DTG results, the main weight loss stage for Nerolidol/HP*β*CD-IC-NF occurred at 356.6 °C, which was significantly higher than the 258.07 °C recorded for nerolidol. Therefore, it was concluded that the thermal stability of Nerolidol/HP*β*CD-IC-NF was superior to that of nerolidol. The TGA and DTG proved the inclusion complex significantly improved the thermal stability of nerolidol.

### 3.6 Phase solubility analysis

**Fig. 4:**
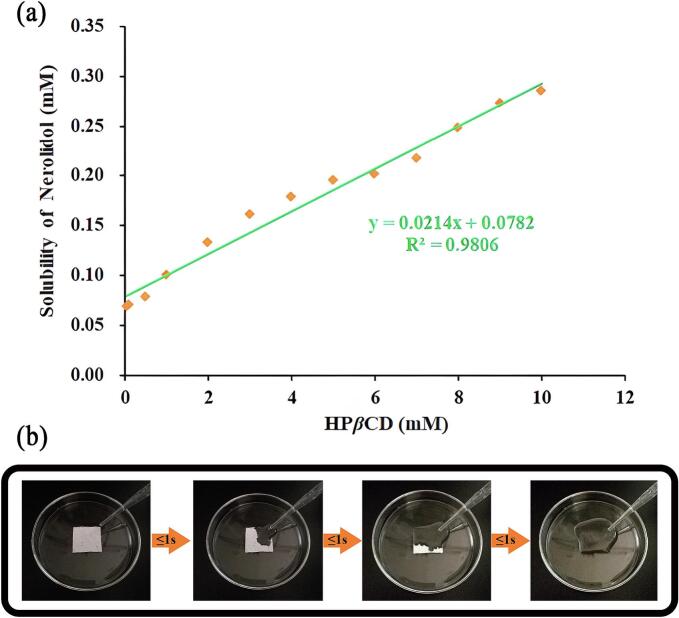
Phase solubility diagram of nerolidol and HP*β*CD (a); Experimental result of fast dissolution of Nerolidol/HP*β*CD-IC-NF (b).

The study of phase solubility can facilitate the optimization of inclusion complexes composition and the analysis of the correlation between nerolidol and HP*β*CD concentrations and their solubility. The phase solubility profiles of nerolidol and HP*β*CD are presented in Fig. 4 (a), revealing a positive correlation between HP*β*CD concentration and nerolidol solubility. According to the early research of Higuchi and Connors (Higuchi & Connors, 1965), the phase solubility diagram exhibited an A_L_-type profile, indicating a stoichiometric ratio of 1:1 between nerolidol and HP*β*CD. Following the formation of the Nerolidol/HP*β*CD-IC, the solubility of nerolidol in 10 mM HP*β*CD increased by a factor of 3.6 compared to its pre-complexation solubility. A higher value of the equilibrium stability constant (*K*s) in the range of 50–2000 M^−1^ indicated the formation of a more stable complex. The *CE* value calculated based on the slope of the phase solubility diagram, providing a more precise description of the binding strength of the complex. In the present study, the *Ks* value of our Nerolidol/HP*β*CD-IC was 279 M^−1^, which was significantly higher than that of Betulin/M*β*CD-IC reported by Zhang et al. (Zhang et al., 2024), which was only 93 M^−1^. This comparative result indicated that the binding between nerolidol and HP*β*CD was more stable, which could effectively improve the water solubility and bioavailability of nerolidol. It further validates the effectiveness of our use of HP*β*CD as the host molecule and enhances its application potential.

### 3.7 Rapid dissolution experiments

To assess the water solubility and dissolution rate of Nerolidol/HP*β*CD-IC-NF, rapid dissolution experiments were conducted. As shown in Fig. 4 (b), the Nerolidol/HP*β*CD-IC-NF was placed in a glass dish, and distilled water was carefully added to the sample. The sample dissolved rapidly in less than three seconds. The transformation of the nerolidol essential oil into an amorphous state upon forming an inclusion complex with HP*β*CD, significantly enhancing the solubility and dissolution rate of nerolidol in water. This improvement expanded the potential applications of nerolidol as an active substance and provide insights into the application of nanofiber membranes.

### 3.8 Molecular simulation

**Fig. 5:**
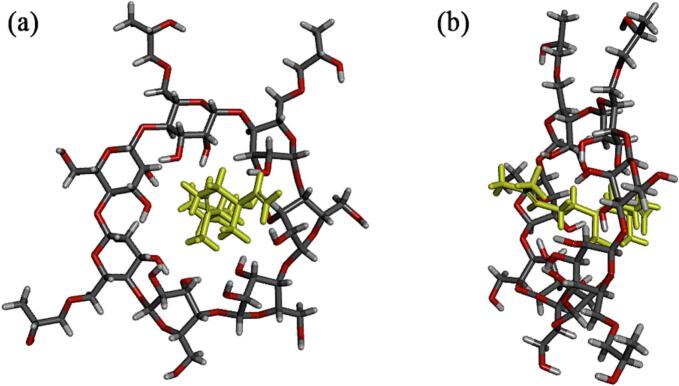
Front view (a) and side view (b) of molecular simulations of nerolidol and HP*β*CD.

Molecular simulation technology enables the prediction of binding affinity and potential binding modes between guest and host molecules, thereby facilitating molecular design optimization and enhancing the performance of inclusion complexes (Burkhanova et al., 2023). As shown in Fig. 5, the front (a) and side (b) views of the minimum energy configuration of the inclusion complex formed by nerolidol and HP*β*CD. The docking results provided feasible docking models, providing the most stable structure of the nerolidol and HP*β*CD binding. The nerolidol were encapsulated in the HP*β*CD cavity, and its methyl group attached to the exposed aldehyde carbon outside the cyclodextrin cavity, which was consistent with the ^1^H NMR results. In the optimal configuration, the binding energy of nerolidol with HP*β*CD was −34.3242 kcal/mol. This negative binding energy suggested that the interaction between the host and the guest released energy, indicating a certain degree of stability in the formation process of the inclusion complex (Li et al., 2025). In addition, the interaction between nerolidol and HP*β*CD involved not only classical hydrogen bonding (conventional hydrogen bonding) but also nonclassical hydrogen bonding (*e.g.*, C—H bonding). The tight binding between the host and guest molecules through the formation of hydrogen bonds confirmed the formation of the Nerolidol/HP*β*CD inclusion complex. In conclusion, through molecular simulation techniques, we have revealed the interaction of the host molecule guest molecule nerolidol with the host molecule HP*β*CD and obtained the most stable binding mode.

### 3.9 Mechanical properties analysis

**Fig. 6:**
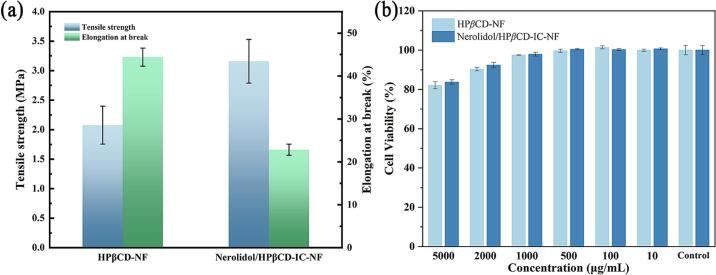
Mechanical properties (a) and cell viability (b) results of HP*β*CD-NF and Nerolidol/HP*β*CD-IC-NF.

The application scenarios of the materials were influenced by the mechanical properties. The mechanical properties of HP*β*CD-NF and Nerolidol/HP*β*CD-IC-NF are shown in Fig. 6 (a). The TS and EAB of HP*β*CD-NF were 2.08 ± 0.32 MPa and 44.40 ± 2.10 %, respectively. After the addition of nerolidol, the TS and EAB of Nerolidol/HP*β*CD-IC-NF were 3.16 ± 0.37 MPa and 22.83 ± 1.30 %, respectively. The increase in TS may be the formation of new hydrogen bonding between nerolidol and HP*β*CD after the addition of nerolidol. However, according to Souza et al. the addition of hydrophobic compounds may disrupt the alignment and continuity of the fiber structure (Souza et al., 2015). This might be the reason for the decrease in EAB. In addition, Hu et al. pointed out that the formulation of inclusion complex nanofibers could be adjusted to meet the requirements of mechanical properties for active food packaging (Hu, Feng, et al., 2024).

### 3.10 Cytotoxicity analysis

The viability of NIH 3 T3 cells cultured with HP*β*CD-NF and Nerolidol/HP*β*CD-IC-NF was tested. For assessing the biocompatibility of nanofibers. As shown in Fig. 6 (b), the survival rates of cells containing nanofibers were all more than 80 %. According to the ISO/EN 10993–5:2009 assessment standard, the material is safe and non-toxic when cell viability exceeds 70 % (Zhao et al., 2024). Therefore, HP*β*CD-NF and Nerolidol/HP*β*CD-IC-NF were considered non-cytotoxic at certain concentrations. Moreover, when the concentrations of HP*β*CD-NF and Nerolidol/HP*β*CD-IC-NF were 100 μg/mL, the cell survival rates were 101.41 ± 0.76 % and 100.44 ± 0.37 %, respectively. This suggested that low concentrations of nanofibers have the potential to promote cell growth.

### 3.11 Antioxidant activity

**Fig. 7:**
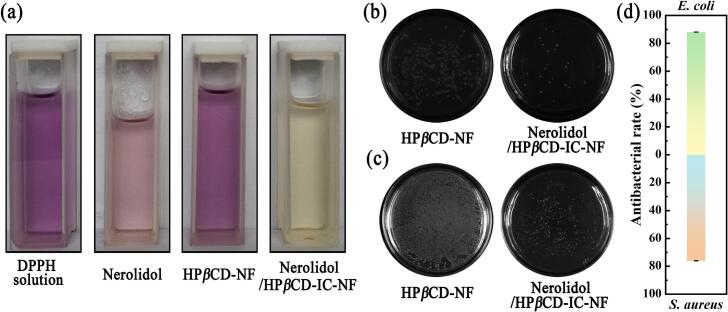
Unreacted DPPH solution, DPPH solution after addition of nerolidol, HP*β*CD-NF, and Nerolidol/HP*β*CD-IC-NF (a); Effectiveness of HP*β*CD-NF and Nerolidol/HP*β*CD-IC-NF in inhibiting *E. coli* (b), and *S. aureus* (c); Antibacterial rate of Nerolidol/HP*β*CD-IC-NF (d).

The antioxidant activity of prepared-nanofiber membranes was evaluated through DPPH radical scavenging experiments, providing important references and evidence for the biomedical and food industries (Bedlovičová et al., 2020). As shown in Fig. 7 (a), the DPPH radical solution stored at room temperature in the dark appears purple. The DPPH radical solution with HP*β*CD-NF as a control group exhibited a similar purple color to the pure DPPH radical solution. Under the same conditions, the DPPH radical solutions with the addition of nerolidol and Nerolidol/HP*β*CD-IC-NF displayed light pink and pale-yellow colors, respectively. According to Feijó de Moura, D. et al. with a TAA percentage of 93.94 % ± 6.06 % (*p* < 0.001), nerolidol possessed more potent antioxidant activity than ascorbic acid (de Moura et al., 2021). The free radical scavenging rate of nerolidol alone was only 20.9 ± 1.8 % as calculated by Eq. (4). The free radical scavenging rates of HP*β*CD-NF and Nerolidol/HP*β*CD-IC-NF were 0.2 ± 0.01 % and 66.4 ± 2.2 %, respectively. Therefore, the antioxidant activity of Nerolidol/HP*β*CD-IC-NF was mainly attributed to nerolidol. In addition, the free radical scavenging rate of the nanofiber membrane was 3.18 times higher than that of nerolidol, which effectively enhanced the application of antioxidant activity of the nerolidol molecule. The experimental colors and calculated results indicated that the Nerolidol/HP*β*CD-IC-NF exhibits significantly enhanced antioxidant activity, which can be ascribed to the improved solubility of nerolidol resulting from IC formation, thereby leading to an overall improvement in its antioxidant potential.

### 3.12 *In vitro* antibacterial test

The viable colony count method is a commonly used microbiological detection technique that allows for the rapid assessment of the antibacterial activity of samples. *E. coli* and *S. aureus* are common bacteria known to cause various diseases in humans and animals. As shown in Fig. 7, the antimicrobial effects of HP*β*CD-NF and Nerolidol/HP*β*CD-IC-NF against *E. coli* (Fig. 7b) and *S. aureus* (Fig. 7c) are illustrated. The antibacterial activities of original HP*β*CD-NF against bacteria were utilized as the control group, while Nerolidol/HP*β*CD-IC-NF was utilized as the experimental group. By comparing the colony counts in the culture dishes, a significant reduction was witnessed in the Nerolidol/HP*β*CD-IC-NF group. Furthermore, the antibacterial rates of Nerolidol/HP*β*CD-IC-NF against *E. coli* and *S. aureus* were calculated to be 88.1 ± 0.2 % and 76.0 ± 0.2 %, respectively using Eq. (5). The results indicates that Nerolidol/HP*β*CD-IC-NF exhibited significant antibacterial activity on both *E. coli* and *S. aureus*. The results of *in vitro* antibacterial experiments coincided with the antioxidant activity results. Excellent antimicrobial properties are an essential requirement for active food packaging and can greatly improve the shelf life of strawberries. Nerolidol was successfully encapsulated in HP*β*CD which was evident from the *in vitro* antimicrobial effect. Meanwhile, the electrospinning process had no effect on the *in vitro* antimicrobial activity of nerolidol.

### 3.13 *In vivo* antifungal test

**Fig. 8:**
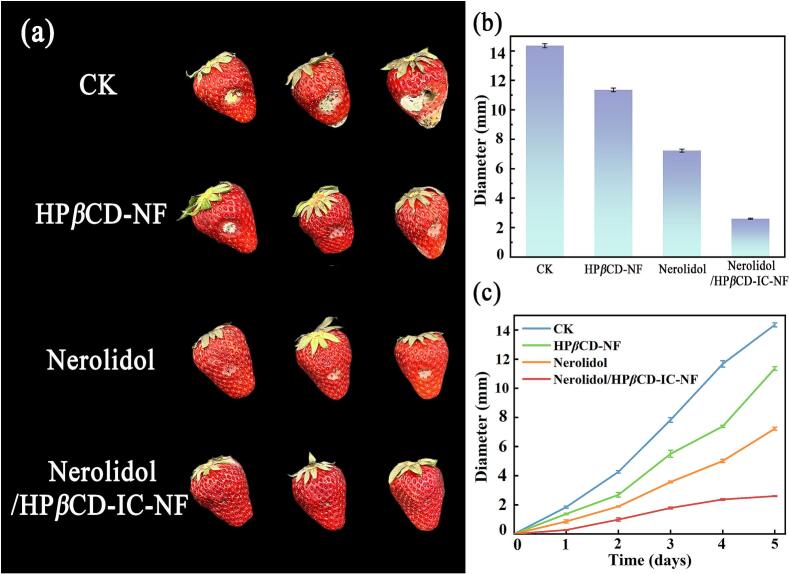
Photographs of strawberry lesions of CK, HP*β*CD-NF, Nerolidol, and Nerolidol/HP*β*CD-IC-NF at the fifth day (a), distribution of lesion diameters (b), and growth of lesion diameters within five days (c).

Strawberries are highly popular due to their nutritional value and high yield. However, they are sensitive to the environment, making them susceptible to spoilage. Fresh strawberries were used to validate the antifungal effect of Nerolidol/HP*β*CD-IC-NF. As shown in Fig. 8, the average diameter of the lesions of blank control strawberries reached 14.36 mm at the fifth day, and the rest of the components had different degrees of growth inhibition of *B. cinerea* on strawberries. Among them, the lesions of strawberry added with nerolidol grew slowly, and its average diameter was 7.11 mm on the fifth day. At the fifth day, the average diameter of strawberry lesions covered by Nerolidol/HP*β*CD-IC-NF was only 2.58 mm, and the lesion was effectively controlled and essentially negligible. There were many studies on strawberry preservation, Zhang et al. reported a nanofiber membrane composed of Zanthoxylum bungeanum essential oil and PVA/*β*-CD complex. The nanofiber membrane provided effective protection for strawberries for more than 10 days, so Nerolidol/HP*β*CD-IC-NF developed in this study has a promising future in the field of fruit packaging (Zhang, Zhang, et al., 2022). Also combined with the *in vitro* antibacterial experiment results, Nerolidol/HP*β*CD-IC-NF showed significant inhibitory effects on both bacteria and fungi. The prepared inclusion nanofiber membranes greatly improved the bioavailability of nerolidol, promoting its potential commercial value in the field of fruit preservation.

### 3.14 Effect of nanofiber membrane on fresh weight and anthocyanin content of strawberries

**Fig. 9:**
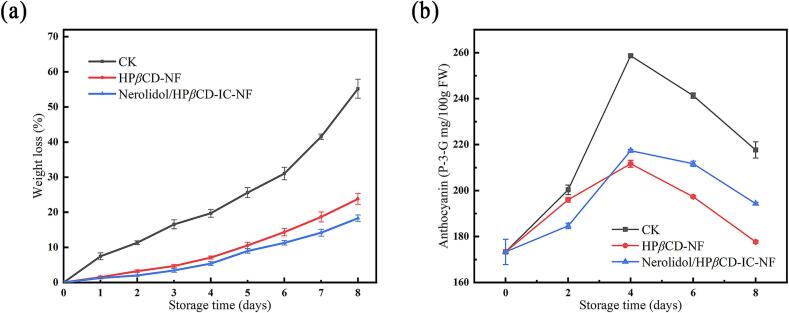
Weight loss (a) and anthocyanin content (b) of strawberries treated with CK, HP*β*CD-NF and Nerolidol/HP*β*CD-IC-NF.

Strawberry weight loss during storage is shown in Fig. 9 (a). The weight of all three groups of strawberries decreased with storage time. The fastest weight loss was observed in the control group, which reached 55.19 % on the eighth day. The weight loss of strawberries was significantly lower in the treatments of HP*β*CD-NF and Nerolidol/HP*β*CD-IC-NF, which were 23.81 % and 18.31 %, respectively. This may be due to the fact that the hydrophilicity of HP*β*CD helps to maintain the circulation of water and gas molecules within the package (Gao, Zhang, et al., 2025). The changes in anthocyanin concentration are shown in Fig. 9 (b). With the increase of storage time, the anthocyanin content of all three groups showed an increasing and then decreasing trend. It is possible that initially strawberries ripened faster and that water loss accelerated anthocyanin release (Fu et al., 2024). Strawberries treated with nanofiber membranes showed no drastic changes in anthocyanin content. It is possible that the inner environment of the package inhibited the respiration of strawberries, which delayed the accumulation of anthocyanins. The Nerolidol/HP*β*CD-IC-NF treatment performed better in anthocyanin retention and effectively slowed down anthocyanin loss due to the continuous uptake of nerolidol (Yin et al., 2024). It helped to maintain the color and nutritional value of strawberries.

## 4 Conclusion

In summary, a novel nerolidol release system Nerolidol/HP*β*CD-IC-NF with uniform, smooth, and bead-free surface is successfully developed by electrospinning technology. Nerolidol was successfully encapsulated within the cavity of HP*β*CD, forming an inclusion complex. The thermal stability of nerolidol in the composite nanofiber was significantly improved compared to that of pure nerolidol. The material showed excellent water solubility in rapid dissolution experiments, so its preservation at high humidity remains challenging. Cytotoxicity experiments demonstrate that the material meets the safety requirements for food packaging. However, its mechanical properties need to be further verified in different application scenarios. Furthermore, the inclusion complex nanofiber membranes exhibited significantly enhanced antioxidant activities. Nerolidol/HP*β*CD-IC-NF was not only effective in controlling bacterial growth, but also in controlling *B. cinerea* on strawberries, expanding its application in fruit preservation. Nerolidol/HP*β*CD-IC-NF lays the foundation for the application of nerolidol in active food packaging. However, due to the cost of electrospinning technology, the feasibility of its large-scale production of nanofiber membranes needs to be further explored.

## CRediT authorship contribution statement

**Yu Zhang:** Writing – review & editing, Writing – original draft, Visualization, Software, Methodology, Investigation, Formal analysis, Data curation, Conceptualization. **Fengrui Li:** Writing – original draft, Visualization, Software, Investigation, Formal analysis, Data curation. **Honglei Yan:** Methodology, Investigation, Data curation, Conceptualization. **Yan Zhang:** Methodology, Formal analysis. **Shimiao Feng:** Data curation. **Lei Deng:** Software. **Lixia Zhao:** Conceptualization. **Shuang Gao:** Writing – review & editing, Supervision, Funding acquisition. **Ying Fu:** Writing – review & editing, Funding acquisition. **Fei Ye:** Writing – review & editing, Funding acquisition.

## Declaration of competing interest

The authors declare that they have no known competing financial interests or personal relationships that could have appeared to influence the work reported in this paper.

## Data Availability

Data will be made available on request.
